# Hemorrhagic shock and encephalopathy syndrome – the markers for an early HSES diagnosis

**DOI:** 10.1186/1471-2431-8-43

**Published:** 2008-10-16

**Authors:** Hiroshi Rinka, Takeshi Yoshida, Tetsushi Kubota, Miho Tsuruwa, Akihiro Fuke, Akira Yoshimoto, Masanori Kan, Dai Miyazaki, Hideki Arimoto, Toshinori Miyaichi, Arito Kaji, Satoru Miyamoto, Ichiro Kuki, Masashi Shiomi

**Affiliations:** 1 Emergency and Critical Care Medical Center, Osaka City General Hospital, Osaka, Japan; 2 Children Medical Center, Division of Pediatric Neurology, Osaka, Japan; 3 Division of Infectious Diseases, Osaka City General Hospital, Osaka, Japan

## Abstract

**Background:**

The hemorrhagic shock and encephalopathy syndrome (HSES) is a devastating disease that affects young children. The outcomes of HSES patients are often fatal or manifesting severe neurological sequelae. We reviewed the markers for an early diagnosis of HSES.

**Methods:**

We examined the clinical, biological and radiological findings of 8 patients (4 months to 9 years old) who met the HSES criteria.

**Results:**

Although cerebral edema, disseminated intravascular coagulopathy (DIC), and multiple organ failure were seen in all 8 cases during their clinical courses, brain computed tomography (CT) scans showed normal or only slight edema in 5 patients upon admission. All 8 patients had normal platelet counts, and none were in shock. However, they all had severe metabolic acidosis, which persisted even after 3 hours (median base excess (BE), -7.6 mmol/L). And at 6 hours after admission (BE, -5.7 mmol/L) they required mechanical ventilation. Within 12 hours after admission, fluid resuscitation and vasopressor infusion for hypotension was required. Seven of the patients had elevated liver enzymes and creatine kinase (CK) upon admission. Twenty-four hours after admission, all 8 patients needed vasopressor infusion to maintain blood pressure.

**Conclusion:**

CT scan, platelet count, hemoglobin level and renal function upon admission are not useful for an early diagnosis of HSES. However, the elevated liver enzymes and CK upon admission, hypotension in the early stage after admission with refractory acid-base disturbance to fluid resuscitation and vasopressor infusion are useful markers for an early HSES diagnosis and helpful to indicate starting intensive neurological treatment.

## Background

Since the original description of the hemorrhagic shock and encephalopathy syndrome (HSES) by Levin et al. [1], numerous cases have been reported in the literature. Although the etiology of HSES remains unknown, this syndrome is associated with acute onset of encephalopathy, shock, watery diarrhea, severe disseminated intravascular coagulopathy (DIC), and renal and hepatic dysfunction.

As some authors have defined the HSES criteria [2-4], patients meeting them will usually have very poor prognoses with a fatal course or severe neurological sequelae. Our experience suggests that early detection of HSES plays an important role in survival and the reduction of neurological sequelae.

## Patients and methods

We described the clinical courses of 8 patients (age range, 4 months to 9 years) who met the HSES criteria of Bacon et al. [3], were admitted to our Intensive Care Unit between November 2001 and August 2007, and whose patient records were reviewed to detect markers for an early diagnosis of HSES.

Patients were excluded if they had an elevated plasma ammonium concentration (>130 μmol/l), historic evidence of Reye's syndrome, inadvertent heating, features of the staphylococcal toxic shock syndrome and/or the hemolytic-uremic syndrome, or if any recognized bacterial pathogens or metabolic disorders were discovered that would explain the illness.

Status epilepticus was defined as an epileptic seizure or seizures lasting more than 30 minutes or recurring within 30 minutes without recovery of consciousness. The biological investigations performed for all 8 patients included white blood cell (WBC) counts, C-reactive protein (CRP), platelet counts, hemoglobin, asparate aminotransferase (AST), and alanine aminotransferase (ALT), creatinine, base excess, creatine kinase (CK), and cell counts of cerebrospinal fluid (CSF). Serum lactate level was measured in 6 patients. DIC was defined as a decreased platelet count and an increase in fibrinogen/fibrin degradation production. Metabolic acidosis was defined as base excess (BE) lower than -3 mmol/L.

Lumbar puncture (LP) was performed upon admission for all patients except one whose LP was performed at the previous hospital and could not be performed at our center because of the CT findings of moderate cerebral edema.

Specimens of blood, urine, stool, sputum, and CSF of all 8 patients were obtained to determine any bacterial and/or viral agents as soon as possible after admission.

Computer tomography (CT) was performed upon admission, electroencephalogram (EEG) was done within 3 hours of admission, and CT was repeated for each patient the following day.

All 8 patients needed mechanical ventilation due to coma and/or seizure. After admission, they required continuous diazepam or barbiturate infusion for seizure or brain edema with the head of the bed elevated 30°.

When hypotension was recognized, fluid resuscitation with a crystalloid or colloid and norepinephrine infusion was set up. The fluid resuscitation target was a central venous pressure (CVP) of ≥ 8 mmHg, urine output >1 ml/kg/hr. Norepinephrine infusion was started when hypotension was refractory with fluid resuscitation. After these cultures were obtained, all the patients took broad-spectrum antibiotics until their bacterial infections were resolved.

The changes of base excess and serum lactate levels were expressed as mean ± SD. When a median was used, the range was given.

The Osaka City General Hospital ethics committee approved this retrospective analysis of patients' data and informed consent was obtained from the patients' next of kin.

## Results

All 8 patients were admitted comatose or with febrile convulsions. Five patients had a history of diarrhea and/or vomiting. The patients' ages ranged from 4 months to 9 years old (median, 1.6 years). Six patients had normal neurological development, however, 2 patients had previously been diagnosed as epileptic. Four patients were admitted in winter, between December and February. Seven patients were transferred to our center within 24 hours after the onset of coma or convulsions. Three patients survived (Table 1).

**Table 1:** Clinical features and outcomes in HSES patients

**Case No**.	**Age yr.mo**	**M/F**	**BT°C**	**History**	**First Sx**	Neurological status*	**Etiology**	**Outcome**
1	9.0	M	41.2	Epilepsy	Pyrexia	Seizure	Unknown	Died
2	1.4	M	39.4	Healthy	Pyrexia	Comatose	Influenza v	MS
3	6.4	F	40.0	Epilepsy	Pyrexia	Seizure	Unknown	Died
4	1.5	F	40.9	Healthy	Diarrhea	Comatose	Unknown	SS
5	1.1	M	39.9	Healthy	Vomiting	Comatose	Norovirus	Died
6	1.8	F	40.0	Healthy	Vomiting	Comatose	Rotavirus	Died
7	0.6	M	42.0	Healthy	Vomiting	Comatose	Adv type 3	Died
8	0.4	F	39.7	LBW	Pyrexia	Seizure	Unknown	SS

Yr, year; mo, month; M, male; F, female; BT, body temperature*; LBW, low birth weight; Sx, symptom; MS; Moderate sequelae; SS, Severe sequelae; v, virus; Adv, adenovirus *Upon admission

All 8 patients had normal platelet counts, blood pressure and a normal or slightly elevated CRP level; 7 patients had a normal hemoglobin level and renal function. All 8 patients had metabolic acidosis and abnormal serum lactate levels. Seven patients had slightly or significantly elevated liver enzymes, CK, and abnormal WBC counts. Bacterial cultures of blood and CSF were negative for all the patients, however, viral pathogens were detected by PCR in 4 patients (Tables 2, 3).

**Table 2:** Laboratory values upon admission 1

**Case No**.	**Platelet ** **10^4^/ul ** (15.0 – 45.0)*	**Hb ** **g/dl ** (11.0 – 14.0)*	**AST/ALT ** **IU/L ** (8–38/4 – 44)*	**Creatinine ** **μmol/L ** (20 – 110)*	**Base excess ** **mmol/L ** (-3 – +3)*	**Lactate ** **mmol/L ** (0.3 – 1.7)*
1	16.5	11.6	109/49	137	-12.7	6.2
2	18.1	10.4	104/27	21	-5.2	--
3	17.4	12.9	122/35	40	-14.2	4.2
4	28.5	12.6	68/14	124	-9.6	--
5	23.5	13.1	204/140	62	-4.4	3.4
6	42.7	11.6	59/31	53	-10.9	11.5
7	21.2	12.5	406/48	84	-16.0	4.2
8	41.1	11.9	207/54	80	-8.5	2.2

Hb, hemoglobin; AST, aspartate aminotransferase;

ALT, alanine aminotransferase; *normal value

**Table 3:** Laboratory values upon admission 2

**Case No**.	**WBC ** **10^3^/ul ** (5.0 – 14.0)*	**CRP ** **mg/dl ** (< 0.5)*	**Shock ** **SBP <70 mmHg**	**CK ** **IU/L ** (55 – 250)*
1	2.8	0.2	-	202
2	9.3	2.0	-	2453
3	17.1	0.1	-	52
4	37.6	0.3	-	907
5	20.5	1.9	-	1241
6	38.2	0.4	-	1070
7	23.1	1.5	-	1144
8	16.4	0.2	-	721

WBC, white blood cell; CRP, C-reactive protein; CK; creatine kinase

SBP, systolic blood pressure; *normal value

Although abnormal cerebral edema was seen in all the patients during their clinical courses, 5 patients appeared normal or only slightly edematous as revealed on their brain CT scans upon admission. On the initial EEG, multifocal paroxysmal discharges were seen in 4 patients, and low-amplitude patterns were seen in 4 other patients. The CSF cell counts were within a normal range in 7 patients, while the serum level of IL-6 and soluble IL-2 receptors increased with varying ranges in all the patients (Table 4).

**Table 4:** CT, EEG features, CSF cell counts and IL levels upon admission

**Case No**.	**Initial CT findings**	**Initial EEG features**	**CSF ** **cell counts/μl ** (< 6)*	**IL-6 ** **pg/ml ** (< 2.6)*	**Soluble ** **IL-2 R U/ml ** (122 – 466)*
1	Slight cerebral edema	MPS	2	2190	2540
2	Slight cerebral edema	MPS	1	6.5	2220
3	Slight cerebral edema	MPS	3	3780	1950
4	Normal	MPS	6	103	827
5	Severe cerebral edema	Low amplitude	3^#^	--	--
6	Moderate cerebral edema	Low amplitude	5	75.4	3320
7	Normal	Low amplitude	3	5344	3360
8	Normal	Low amplitude	5	-	1030

CT, computer tomography; EEG, electroencephalogram; CSF, cerebrospinal fluid; IL, interleukin; R, receptor; MPS, multifocal paroxysmal discharges

^#^Performed at a previous hospital; *normal values

All 8 patients had hemodynamic failure within 24 hours after being admitted; therefore, fluids were infused to maintain arterial pressure with the range of fluid balance from -6 to 275 ml/kg (median, 61 ml/kg) for 24 hours from admission for hypotension. Norepinephrine was given to all patients ranging from 0.1 to 0.5 μg/kg/min (median, 0.3 μg/kg/min). Twenty-four hours after admission, 6 patients had normal renal function, and 4 patients had normal platelet counts. However, 5 patients exhibited a decrease in hemoglobin (Table 5).

**Table 5:** Laboratory values 24 hours after admission and hemodynamic therapy

**Case No.N.V**.	**Platelet ** **(10^4^/ul) ** **(15.0–45.0)**	**Hb ** **(g/dl) ** **(11.0–14.0)**	**AST/ALT ** **(IU/l) ** **(8–38/4–44)**	**Creatinine ** **(μmol/L) ** **(20–110)**	**BE ** **(mmol/l) ** **(-3–+3)**	**Lactate ** **(mmol/L) ** **(0.3–1.7)**	**FB ** **(ml/kg/24 h)**	**NE ** **(μg/kg/min)**
1	15.2	12.0	293/162	192	-6.5	6.6	77	0.1
2	24.0	9.8	130/39	20	-4.2	--	0	0.1
3	1.6	8.4	550/62	90	-7.2	5.8	275	0.5
4	9.4	12.2	198/36	133	-5.1	--	66	0.5
5	18.1	10.7	58/42	19	-3.1	2.1	56	0.5
6	33.7	10.0	65/40	37	-4.6	10.8	-2	0.5
7	4.6	10.0	841/341	75	-8.3	6.8	187	0.1
8	9.5	9.4	233/78	35	-3.8	2.1	-6	0.1

Hb, hemoglobin; AST, aspartate aminotransferase; ALT, alanine aminotransferase; BE, base excess; FB, fluid balance; NE, norepinephrine; N.V., normal values

**Figure 1:**
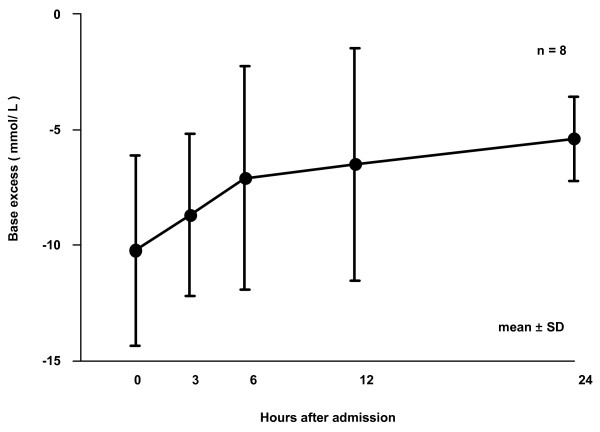
Time course of base excess for the first 24 hours after admission.

All the patients exhibited a severe metabolic acidosis with the BE range from -16.0 to -4.4 mmol/L (median, -10.3 mmol/L) upon admission. The acid-base disturbances were maintained with the BE range from -14.4 to -4.1 mmol/L (median, -7.6 mmol/L) at 3 hours, and from -15.2 to -3.1 mmol/L (median, -4.7 mmol/L) at 12 hours after admission with infusion of fluids and/or norepinephrine. The metabolic acidosis was refractory to intensive treatment with mechanical ventilation, infusion of fluids and/or norepinephrine at 24 hours with the BE range from -8.3 to -3.1 mmol/L (median, -4.9 mmol/L; Figure 1). Sodium bicarbonate for metabolic acidosis was not administered because the blood pH was kept in the normal range (7.35–7.45) with effective ventilation.

Similarly, there was a tendency to maintain the elevated serum lactate levels, which were measured in 6 patients, with the range from 2.2 to 11.5 mmol/L (median, 4.2 mmol/L) upon admission, from 2.3 to 11.8 mmol/L (median, 6.0 mmol/L) at 12 hours after admission, and from 2.1 to 10.8 mmol/L (median, 6.2 mmol/L) at 24 hours after admission.

**Figure 2:**
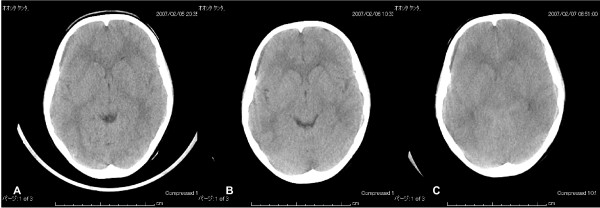
**The changes of CT findings at the level of the basal ganglia of Case 1**. A: CT scan on admission showing slight edema without loss of gray or white matter differentiation. B: CT scan 24 hours after admission showing definite basal cistern and cerebral sulci. C: CT scan 48 hours later showing severe edema with vanishing basal cisternae and loss of gray and white matter differentiation.

CT scan revealed abnormalities between 24 and 72 hours after onset of coma or seizures (Figure 2). When the CT revealed bilateral cortical and subcortical areas of low density, those patients had DIC, anemia and multiple organ failure. Nevertheless, the respiratory function was maintained during the clinical course of all patients.

**Figure 3:**
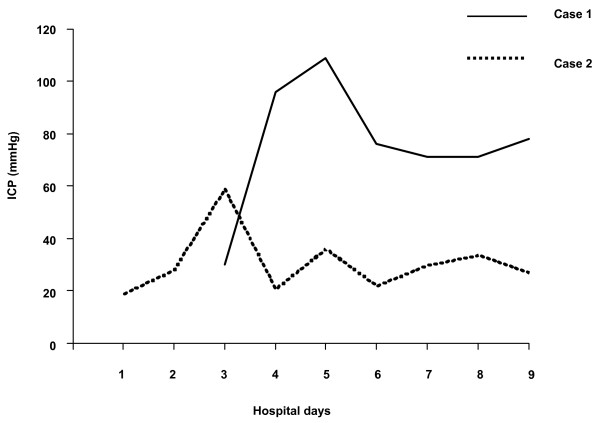
**Time course of ICP in Cases 1 and 2 after admission**. ICP control was difficult when monitoring was started after the abnormal CT finding was discovered in Case 1, therefore, the maximum ICP was increased to 109 mmHg. ICP monitoring started before the presence of abnormal CT findings in Case 2 in which the maximum ICP was increased to 59 mmHg; however, the CPP could be maintained above 50 mmHg. This patient was the only case with mild sequelae among all our cases.

We started intracranial pressure (ICP) monitoring (REF 110-4BT, Camino, USA) in Cases 1 and 2. In Case 1 the ICP monitoring was started from when the abnormal CT finding was discovered; however, in Case 2, it was started immediately after admission, i.e., before an abnormal CT finding. The ICP was controlled with whole-body hypothermia (34° – 34.5°C) with barbiturate infusion and osmolar diuretics. When the ICP had increased to over 30 mmHg against all our efforts, we increased the rate of norepinephrine infusion to maintain the cerebral perfusion pressure (CPP). However, it was difficult to control the ICP and CPP in both of these cases. The maximum ICP was 109 and 59 mmHg in Cases 1 and 2, respectively (Figure 3). Patient 1 died on hospital day 24; however, patient 2 improved and was transferred to the pediatric ward on hospital day 15 as the only patient with moderate sequelae among all 8 patients.

## Discussion

In 1983, Levin et al. described a devastating disease occurring in early childhood called the hemorrhagic shock and encephalopathy syndrome [1]. HSES is not a common disease. Sofer et al. reported 20 patients diagnosed with HSES in a population of about 400,000 over an 11-year period [5]. Our center at Osaka City General Hospital is one of two tertiary pediatric centers in Osaka prefecture. The area our center services for primary referrals has a population of about 4 million residents. However, we do not in any way hypothesize that the 8 patients in our 6-year study were the only cases of HSES in this large region, as compared with the 20 patients in the 11-year Sofer et al. report of a significantly smaller population [5], as there were most likely other HSES patients who could not be transferred to our center from other hospitals due to the rapid deterioration of their conditions. Most reported cases of HSES occurred in winter [3,5]; as in the present study, further supporting this evidence, 50% of our patients were admitted in winter, between December and February.

The outcome of HSES was often fatal or with severe neurological sequelae [2-6]. In the present study, 5 patients died, and the 3 survivors had neurological sequelae. The HSES criteria have been defined in previous reports [2-4]. The clinical presentation includes shock, coma and/or seizure, hemorrhage, diarrhea, and oliguria. Laboratory investigations reveal decreased hemoglobin and platelet counts, evidence of DIC, elevated creatinine, AST and ALT, and metabolic acidosis. However, when patients met the HSES criteria, their condition was always critical with multiple organ failure. We determined that these HSES criteria were not useful to help develop an adequate treatment for patients with HSES.

In these cases, the first problem the physician is faced with is the difficulty of the differential diagnosis. Some reports have indicated in the differential diagnosis the diseases of the toxic shock syndrome, the hemolytic-uremic syndrome, and Reye's syndrome, among others [6-8]. In our patients, those diseases were excluded because of the lack of skin or mucosal manifestations, hemolysis, and/or blood ammonia levels. The clinical course of our patients was not indicative of any of these diseases. Heatstroke has similar clinical and pathological features to HSES; however, there was no history of over wrapping or excessive heating in any of our patients in the present study.

The most common first symptoms were seizure or coma following fever in our cases. From our experience, we have found that common febrile convulsion is often the most difficult disease in the differential diagnosis of HSES in the early stage. Concerning febrile convulsions: prolonged seizures lasting 5 to 10 minutes are relatively common, the laboratory data including CSF is near normal, CT scans reveal normal or slightly edematous conditions, and elevation of CK levels and WBC counts are often seen in cases with prolonged seizures [9-12]. Even though follow-up CT scans could provide useful information about cerebral edema [13-15], our results showed that the initial CT finding was not useful in making the HSES diagnosis. When an abnormality on the CT was discovered, the patient's condition was critical.

Harden et al. have reported the importance of EEG features and evolution [16], as confirmed by the observations in the present study, the initial EEG features appeared abnormal in all patients. However, in the initial EEG features, it is usually difficult to distinguish HSES from other diseases with convulsions including febrile convulsions. Rosman has reported that the initial EEG features in patients with febrile convulsions are abnormal in as many as 88% of the patients [17].

Dunn reported that the outcome of the status epilepticus was not associated with acidosis on admission [9]. Imuekemhe et al. reported that mean serum lactate on admission was significantly higher in patients with prolonged febrile convulsions compared to the corresponding mean value in patients with only brief convulsions [18]. Conversely, Levin et al. noticed metabolic acidosis in HSES patients upon admission [2]. Ince et al. also reported that metabolic acidosis was the common laboratory value in HSES patients upon admission [15]. Little et al. reported a marked metabolic acidosis being refractory to fluid-resuscitate in HSES patients [6]. And Idro et al. reported that the level of base excess of < -8 mmol/l was a prodromal risk factor for death among children with acute seizures [19].

In the present study, deterioration of the patients' conditions, especially hemodynamic failure, was dramatic up to 24 hours after admission. The most effective treatment for metabolic acidosis with high levels of serum lactate is the adequate and timely treatment of fluid resuscitation and vasopressor [20,21]. All patients needed large amounts of fluids and/or norepinephrine infusion. The median rate of fluid administration needed was 61 ml/kg for 24 hours with the infusion of norepinephrine. However, neither metabolic acidosis nor abnormal serum lactate improved in this study.

Sepsis from bacterial infection was excluded by the negative bacterial cultures and the normal CRP levels. However, the hemodynamic course of our patients was very similar to severe septic shock.

The etiology of HSES is still unknown. It has been reported that cytokine storm may be associated with the progress of acute encephalopathy including HSES [22-24] as septic shock, and levels of some cytokines were useful markers for HSES [25,26]. The serum levels of IL-6 and soluble IL-2 receptors were increased in our patients, however, the degree of the increase varied in each patient. These results suggest that the increase in cytokines may be associated with HSES. As septic shock is characterized by severe vascular leakage, this is the reason that large amounts of fluids and/or norepinephrine infusions were needed for the patients in this study.

As previously expressed, the respiratory functions of the HSES patients in the present study were maintained throughout their clinical courses. This is the most essential difference between HSES and sepsis in the state of multiple organ failure. Sepsis-induced acute lung injury (ALI)/acute respiratory distress syndrome (ARDS) has been reported [27], and the inflammatory response in ALI/ARDS is associated with the release of cytokines [28]. A limitation of the present study was its small population of only 8 children. Therefore, we did not find a definitive reason the cytokine storm with HSES did not have a significant influence on the pulmonary vascular permeability, as would a prodromal marker of ALI/ARDS. It is our supposition that this difference is a key point that would detect the etiology of HSES.

Further studies are warranted to discover what kinds of cytokines would be the most useful markers. The current problem that we face in our center is that it takes considerable time and effort to get the necessary results for these cytokines.

We detected viral pathogens in 4 of 8 patients in the present study. Viral infection may be the trigger for the pathogenesis of HSES as reported in the case report by Gooskens et al. [22]. It may be associated with the most common season of HSES – winter.

Even though there were abnormalities in coagulation, hemoglobin, and renal function, they were useful to make a diagnosis of HSES, it was evident that these laboratory abnormalities were not appreciated as diagnostic markers until at least 24 hours after admission [14,29].

Therefore, we did not have sufficient time to make a proper diagnosis of HSES using the established criteria [2-4]. A diagnosis of HSES ought to be made within 24 hours of admission, otherwise the patients' conditions worsen and the window to provide adequate treatment closes.

The second problem is the treatment of patients with HSES. Even though prolonged metabolic acidosis and/or high levels of serum lactate refractory to large amounts of fluids and/or norepinephrine infusion are useful markers for an early diagnosis, they are not useful as markers when only respiration and circulation management is provided. Because the brain appears to be the main target organ of HSES, in our non-surviving patients, severe diffuse brain edema with loss of differentiation between the gray and white matter was found on the CT scan during the clinical course. And similar CT abnormalities have been described in non-surviving patients in other reports [13,15].

The cause of brain edema following HSES remains unclear, however, Unterberg et al. reported that brain edema of traumatic brain injury was associated with various mediators including cytokines, lactate, free oxygen radicals, etc. [30]. Brain edema in HSES patients seems to occur in such a situation. Because the serum levels of cytokines and lactate had increased, an increase in vascular permeability was suggested by our patients needing large amounts of fluids. Furthermore, large amounts of fluid-resuscitate within 24 hours of admission may lead to brain edema under the state of increasing vascular permeability.

We propose that for the most efficacious management of ICP, whenever possible, ICP monitoring ought to be started before detection and observation of any decrease in the platelet count and/or any abnormal CT findings because ICP monitoring could not be performed with DIC. This is the reason that a diagnosis of HSES should be made within the early stage, i.e., within 24 hours of admission.

However, to our knowledge, there have not been any reports published proposing an effective treatment for HSES. Controlling brain edema might be the optimal therapy to help HSES patients survive. Even though there are currently only palliative therapies, e.g., mild hypothermia, infusion of fluids and osmotic diuretics, administration of anticonvulsants under mild hyperventilation, and vasoconstrictor infusion to prevent edema and to maintain the cerebral perfusion pressure (CPP). There are no reports about the efficacy of the control of ICP and CPP upon the outcome of the HSES; however, we suggest that the control of ICP and CPP is an essential part of any therapeutic treatment.

## Conclusion

A patient characterized by coma or seizure following hyperpyrexia might be diagnosed as having common febrile convulsions. However, when such a patient also presents with elevated liver enzymes and CK upon admission, hypotension within 24 hours after admission, with refractory acid-base disturbance and an abnormally high serum lactate level, even with fluid-resuscitate and/or vasopressor infusion, these signs may be useful markers for an early HSES diagnosis and indicators to start intensive neurological treatment. HSES is not a disease that can be diagnosed easily using the current diagnostic criteria, however, HSES can be predicted in the early stage of its clinical course using these new prodromal diagnostic markers.

## Key messages

• When the patients met the HSES criteria, their condition was always critical with multiple organ failure.

• CT scan, DIC, EEG, or renal function upon admission did not prove useful for an early diagnosis of HSES.

• Elevated liver enzymes and CK upon admission, hemodynamic failure in the early stage after admission, and a prolonged metabolic acidosis refractory to intensive treatment were useful markers for an early diagnosis of HSES.

• Controlling brain edema might be the most important therapy to help HSES patients survive.

• HSES is a disease that should be predicted within the early stage of its clinical course.

## Abbreviations

ALI: acute lung injury; ALT: alanine aminotransferase; ARDS: acute respiratory distress syndrome; AST: aspartate aminotransferase; BE: median base excess; CK: creatine kinase; CPP: cerebral perfusion pressure; CRP: C-reactive protein; CSF: cerebrospinal fluid; CT: computed tomography; CVP: central venous pressure; DIC: disseminated intravascular coagulopathy; EEG: electroencephalogram; HSES: hemorrhagic shock and encephalopathy syndrome; ICP: intracranial pressure; LP: lumbar puncture; WBC: white blood cell.

## Competing interests

The authors declare that they have no competing interests.

## Authors' contributions

HR conceived and designed the study, wrote the manuscript, and was responsible for all stages of the study. TY, AY, HA, TM and IK were the attending doctors for the patient, participated in the study design, were responsible for collection of the data. TK, MT, AF and DM participated in the study design, collected and interpreted the data and provided suggestions for analysis. AK, SM and MS provided critical revision of the manuscript for important intellectual content and helped to draft the manuscript. MK set the ICP monitoring in two patients as a neurosurgeon, analyzed the results and participated in its design. All authors read and approved the final manuscript.

## Pre-publication history

The pre-publication history for this paper can be accessed here:


